# Telehealth delivery of physical therapist-led interventions for persons with chronic low back pain in underserved communities: lessons from pragmatic clinical trials

**DOI:** 10.3389/fpain.2024.1324096

**Published:** 2024-04-19

**Authors:** Julie M. Fritz, Isaac Ford, Steven Z. George, Laura Vinci de Vanegas, Tyler Cope, Colleen A. Burke, Adam P. Goode

**Affiliations:** ^1^ Department of Physical Therapy & Athletic Training, University of Utah, Salt Lake City, UT, United States; ^2^ Department of Orthopaedic Surgery, Duke University School of Medicine, Durham, NC, United States; ^3^ Duke Clinical Research Institute, Duke University School of Medicine, Durham, NC, United States; ^4^ Department of Population Health Sciences, Duke University School of Medicine, Durham, NC, United States

**Keywords:** pragmatic clinical trials, telehealth, health equity, chronic pain, physical therapy

## Abstract

In this perspective, we present our experience developing and conducting two pragmatic clinical trials investigating physical therapist-led telehealth strategies for persons with chronic low back pain. Both trials, the BeatPain Utah and AIM-Back trials, are part of pragmatic clinical trial collaboratories and are being conducted with persons from communities that experience pain management disparities. Practice guidelines recommend nonpharmacologic care, and advise against opioid therapy, for the primary care management of persons with chronic low back pain. Gaps between these recommendations and actual practice patterns are pervasive, particularly for persons from racial or ethnic minoritized communities, those with fewer economic resources, and those living in rural areas including Veterans. Access barriers to evidence-based nonpharmacologic care, which is often provided by physical therapists, have contributed to these evidence-practice gaps. Telehealth delivery has created new opportunities to overcome access barriers for nonpharmacologic pain care. As a relatively new delivery mode however, telehealth delivery of physical therapy comes with additional challenges related to technology, intervention adaptations and cultural competence. The purpose of this article is to describe the challenges encountered when implementing telehealth physical therapy programs for persons with chronic low back pain in historically underserved communities. We also discuss strategies developed to overcome barriers in an effort to improve access to telehealth physical therapy and reduce pain management disparities. Inclusion of diverse and under-represented communities in pragmatic clinical trials is a critical consideration for improving disparities, but the unique circumstances present in these communities must be considered when developing implementation strategies.

## Introduction

Chronic low back pain (cLBP) is among the most common reasons for healthcare visits (1, 2) and results in greater financial costs and loss of quality of life than any other chronic health condition (3, 4). The burden of cLBP falls disproportionately on persons in underserved communities. Persons with few socioeconomic resources, living in rural communities, who are Veterans, or part of racial/ethnic minoritized groups are at greater risk of experiencing chronic pain, especially chronic pain that results in substantial restriction of daily activities and the ability to work (5).

Disparities related to cLBP are evident in access to evidence-based care. Guidelines advocate first-line nonpharmacologic care emphasizing active coping strategies including physical activity, patient education, and exercise many of which are provided by physical therapists (PTs) (6). Guidelines advise against low-value services that promote passive coping including rest and opioid therapy (6–8). Practice patterns, however, reveal persistent evidence-practice gaps with over-utilization of opioids and underuse of nonpharmacologic therapy (9). Gaps are particularly notable for persons from racial or ethnic minoritized communities, those with fewer economic resources, living in rural areas and Veterans receiving care outside of large, urban Veteran's Administration (VA) facilities. Persons in these communities are less likely to receive nonpharmacologic interventions and are often more likely to receive opioids for cLBP (10–13).

Persistent evidence-practice gaps for persons with cLBP has motivated interest in pragmatic clinical trials (PCTs) to increase use of nonpharmacologic interventions. PCTs are designed to examine effectiveness of interventions under real-world circumstances (14). As such, PCTs should include diverse, heterogeneous patient populations that accentuate generalizability. Prior to COVID, PT-led telehealth delivery of nonpharmacologic interventions was discussed as a strategy to overcome access barriers, but was studied sparingly (15, 16). Research interest increased substantially with COVID (17). however, most studies have not included patients from communities that experience pain management disparities (18).

The authors of this paper are conducting PCTs examining PT-led telehealth interventions for persons with cLBP who are from communities that experience disparities. Our purpose is to describe the challenges experienced conducting PCTs evaluating PT-led telehealth interventions for persons with cLBP in underserved communities; and discuss strategies to overcome these challenges in order to reduce pain management disparities.

### Pragmatic clinical trials being conducted in underserved communities

The authors are conducting two PCTs examining PT-led telehealth interventions in communities that experience disparities. The Improving Veteran Access to Integrated Management of Back Pain (AIM-Back) trial is examining two care pathways for LBP in multiple VA Medical Center sites (19). Veterans are more likely to experience chronic pain (20), and to be impacted by multiple chronic health conditions and psychological distress than non-Veterans (21, 22). Veterans who receive care in the VA Health System are more likely to live in rural communities, be from racial/ethnic minoritized communities, and have income below the federal poverty level relative to Veterans who receive care outside the VA (23, 24). The AIM-Back trial recruited VA sites that include smaller clinics without an existing relationship to an academic institution or a VA Center for Innovation site, resulting in 19 geographically-distinct clinics of which 10 are community-based clinics not connected to a larger VA facility. This approach enhanced the inclusion under-represented populations by engaging VA sites typically left out of clinical research.

BeatPain Utah is evaluating two strategies to provide telehealth PT for adults with cLBP who receive care in Community Health Centers (CHCs) in Utah (25). CHCs are federally-funded, non-profit organizations providing primary care in areas with high prevalence of medically underserved individuals (26). Nationally, about 1 in 12 Americans, including 1 in 5 residents of rural communities, receive primary care in a CHC, a majority of whom are members of racial/ethnic minoritized groups and have a household income below the federal poverty level (27, 28). The prevalence of chronic pain is 40%–60% among adult CHC patients (29, 30). In Utah, half of the state's 60 CHC clinics operate in rural or frontier counties, with 49% of patients served identifying their ethnicity as Hispanic/Latino, 39% best served in a language other than English, 45% are uninsured, and 59% have a household income below the federal poverty level (31).

### Use of physical therapist-Led telehealth interventions in AIM-Back and BeatPain Utah PCTs

The goal of the AIM-Back trial is to compare the effectiveness of two pathways to improve access to nonpharmacologic pain care. One pathway uses a local pain navigator to assists veterans to navigate non-pharmacological care options within their VA system. The alternative pathway involves integrated sequenced care that provides an in-person PT visit followed by weekly telehealth sessions for 6 weeks; creating a hybrid approach. Telehealth sessions use two-way video or by phone and focus on physical activity counseling. After 6 weeks Veterans return to in-person PT and complete a risk stratification screening (32). Veterans at medium or high-risk for prolonged disability receive an additional 6 weeks of phone-based, telehealth PT including psychological and behavioral activation components such as pain coping skills and behavioral techniques.

The goal of the BeatPain trial is to compare the effectiveness of two strategies to provide telehealth PT using brief or extended delivery. Both strategies are completely remote with no in-person contact. The two strategies provide either 2 or 12 telehealth sessions in treatment phase I. At the conclusion of phase I all patients are evaluated. Those initially receiving 2 sessions are provided 10 additional sessions if they are determined to be non-responders to the initial 2 sessions. Those receiving 12 sessions do not receive additional treatment. Patients are referred to BeatPain PTs by primary care providers in participating CHCs using an electronic referral process. Telehealth sessions are provided in English or Spanish based on patient preference and use synchronous video or phone-based communication.

Both BeatPain and AIM-Back deliver care to patients in underserved communities typically omitted from clinical trials. Both studies are using telehealth to provide PT care that involves physical activity, exercise, education and psychologically-informed strategies. Both studies gathered input from community members and have learned lessons as the trials proceeded. We grouped issues encountered into four areas to characterize challenges encountered in providing PT-led telehealth interventions. These issues are outlined in Table 1 and described below.

**Table 1:** Overview of barriers and facilitators encountered in the development and implementation of physical therapist-led telehealth interventions and the strategies used to deliver telehealth interventions in the BeatPain and AIM-Back pragmatic clinical trials.

	Barriers in underserved communities	Facilitators in underserved communities	Strategies
Patient Access Considerations	Patients work multiple jobs with less predictable hours	Most people in underserved communities have mobile phones that can be used for sessions.	Offer sessions outside regular working hours
	Multi-generational homes and housing instability make it challenging to find space for telehealth		Be flexible and non-judgmental in scheduling/re-scheduling sessions
		Being flexible and willing to work with a patient's availability builds trust with the physical therapist	
			Attempt to have same physical therapist work with a patient throughout treatment
	Internet access and technology may be limited, restricting sessions to audio-only delivery		
Physical Therapy Intervention Adaptations	Community resources for exercise and physical activity can be limited	Many mHealth resources are available to support patients	Use exercise and physical activity interventions with simple instructions that are personalized to the patient
		Interventions that promote active pain coping strategies such as relaxation techniques or mindfulness are evidence-based and not resource or equipment dependent	
	Patients are less likely to be physically active		
	Patients are more likely to experience emotional and financial stressors, have a high co-morbidity burden		Integrate mHealth resources to support patient education and exercise programs
			Include cognitive behavioral techniques to address stress and resiliency factors
Patient—Therapist Working Alliance	Creating an effective patient-therapist alliance while using telehealth can be more difficult	Effective communication can build working alliance even when using telehealth	Training physical therapists to provide care using a combination of motivational interviewing and cognitive behavioral therapy
	Enhancing motivation for behavior change is challenging	Motivational interviewing is an effective communication strategy to build self-efficacy for behavior change using telehealth	
	Language, culture and health literacy may further challenge the patient-therapist alliance		
Culturally-Competent Care	Patients in underserved communities are more likely to have cultural backgrounds that differ from their therapist.	Providing care that respects the cultural background of patients helps build trust with the physical therapist	Training physical therapists in cultural competencies including awareness of their own cultural background.
		Motivational interviewing may be especially beneficial for supporting persons in underserved communities and reducing potential for therapist bias.	Providing care using a combination of motivational interviewing and cognitive behavioral therapy to promotes patient-centered care.
	Therapists may have misconceptions or implicit biases based on patient's cultural background		
	Patients may have pain beliefs and coping preferences based on cultural background that are unhelpful for recovery.		

### Patient access considerations

Telehealth overcomes barriers related to geographic proximity and transportation, but does not eliminate other access barriers contributing to disparities. Persons in underserved communities are more likely to have multiple persons living in the same household, experience housing instability, and are more likely to hold multiple jobs with irregular work hours (33); creating challenges in finding time and space for telehealth sessions. The ability to offer services outside traditional working hours and scheduling flexibility can be essential for telehealth access. If other people are present it is important to ask and document the patient's approval for another person to be present.

The digital divide, which relates to digital literacy and availability of technology for telehealth (34), can pose additional access barriers. Adults in rural and racial/ethnic minoritized communities are less likely to own a tablet or traditional computer (35). Access to broadband internet is a barrier in rural communities, where about a quarter of residents find high speed internet access a major problem, a figure that increases to 31% for non-white residents in rural communities (35). Low digital literacy impacts access when persons lack experience setting up web cameras, accessing software, downloading apps (36), and the overall cognitive load of learning new tasks with unfamiliar technologies (37). Collectively these factors make it more likely that persons in underserved communities will rely on their phone for audio-only telehealth instead of 2-way video communication (38). While audio-only telehealth was common during COVID, using the phone instead of video communication has been associated with lower patient satisfaction (39), and may be particularly challenging for providing PT interventions.

In the BeatPain study we addressed access by ensuring PTs are available during early evening and morning hours. We adopted a flexible work schedule for PTs and hired people available at different times. The PT training emphasizes the need for flexibility, recognizing that participants' time limitations may require shorter sessions. Sessions may need to be rescheduled with short notice so PTs keep times open during the week to permit timely rescheduling. It is important that these circumstances be accommodated in a patient-centered, non-judgmental manner. Every effort is made to have the same PT provide care for a BeatPain participant throughout the study as provider consistency can help reduce missed appointments (40).

The AIM-Back study facilitated access by taking advantage of resources available through the VA health system; a leader in expanding telehealth access. Veterans and providers can use VA Video Connect, a secure videoconferencing app, for telehealth sessions on a smartphone, tablet, or computer. However, approximately 15% of Veterans do not have internet access (41), and many, including rural or low income Veterans, lack access to necessary technology or connectivity. As such, AIM-Back telehealth providers educate Veterans on possible services offered by the VA to improve telehealth access including Digital Divide Consults that help Veterans obtain services necessary for telehealth, including lending internet-connected tablets and eliminating data charges for Veterans using the VA Video Connect app. Accessing Telehealth through Local Area Stations (ATLAS) provides Veterans with private space in their local community to use for video appointments (42).

### Physical therapy intervention adaptation

Exercise is an evidence-based strategy for persons with cLBP and a core component of PT practice (43). Persons in underserved communities are less likely to have access to spaces conducive to physical activity, and are less likely to be physically active (44–46). Veterans and persons in rural communities are more likely to have co-morbidities including mental health conditions, diabetes, and obesity that can pose additional challenges for exercise (46–48). These factors must be considered in developing PT-led telehealth interventions. Additionally, emotional and financial stressors are risk factors for high impact cLBP and are prevalent among Veterans and persons in low income communities (49, 50). Addressing these stressors through coping strategies such as relaxation techniques, sleep quality, mindfulness and countering negative cognitions around pain may be less familiar to PTs (51), but these techniques can be effective in offsetting the impact of cLBP on daily life (52), can be effectively delivered by PTs (53, 54), and are amenable to telehealth delivery.

The BeatPain and AIM-Back studies considered these factors when developing their telehealth PT protocols. Emphasis is placed in both studies on simple exercise activities such as walking that can be explained to participants even when care is audio-only. Exercise programs are supplemented with Mobile Health (mHealth) applications for asynchronous instruction including videos or written performance descriptions. BeatPain uses Medbridge (Medbridge, Inc., Bellevue, WA) to create personalized programs that include videos, audio, handouts and pictures that patients can access asynchronously through a mobile app, or emailed or texted to a patient. AIM-Back provides Veterans with informational packets that include all prescribed exercises with picture demonstrations and detailed text describing technique, frequency, and intensity. Materials for the psychologically-informed components include education materials on behavioral interventions and handouts to increase engagement from Veterans and encourage active participation in treatment. These materials are also made available digitally, through the MyHealtheVet portal or email. Both AIM-Back and BeatPain PT integrate cognitive and behavioral strategies that focus on reducing maladaptive cognitions about pain including kinesiophobia and catastrophizing that may reduce engagement in physical activity (55). Strategies to reduce these cognitions include education, reframing, goal setting and graded exposure, problem-solving, activity pacing and relaxation techniques (e.g., visualization, mindfulness, pain journaling, etc.). These strategies were also supported by supplemental print materials and videos available to participants.

### Patient—therapist working alliance

The working alliance, or therapeutic bond, between patient and PT is an integral component of engagement and behavioral change, and predicts outcomes of in-person PT care (56, 57). Developing an effective working alliance can be challenging when care is provided using telehealth as patient surveys suggest the care may be perceived as less personal (58, 59), particularly with audio-only delivery (60). Surveys of PTs providing telehealth highlight concerns about developing rapport without the ability to touch, or perhaps see, their patient (61). Concepts contributing to effective patient-PT working alliances include attention to the patient's needs, understanding their narrative through active listening, providing a safe therapeutic space for patients to set meaningful goals and build autonomy (60, 62). These concepts can be effectively developed using telehealth, but likely require additional skills for PTs whose experience has primarily involved in-person delivery.

In BeatPain, PTs are trained to integrate motivation and problem-solving (MAPS). MAPS is an approach to enhance patients' intrinsic motivation for behavior change that combines motivational interviewing (MI) and cognitive behavioral practices (63), that has been found effective for engaging patients in behavior changes for smoking cessation and substance use (64–66). MAPs was considered apposite for BeatPain because it uses MI, a patient-centered communication strategy that facilitates active listening and is well-suited to audio-only delivery (67, 68). Problem-solving techniques used with MAPS are intended to enhance intrinsic motivation for change by guiding patients to develop personalized goals and build self-efficacy; an important factor for limiting the functional impact of LBP (69). MAPS emphasizes expressions of empathy for PTs through active, nonjudgmental listening about the patient's perspective and goals (65). Expressing empathy in the context of telehealth can be challenging, but is important for developing an effective patient-PT working alliance (70, 71). Additional efforts to enhance empathy included minimizing distractions and background noise during sessions and encouraging picture-in-picture functions during video telehealth sessions when possible to allow eye contact.

Similar strategies were used in the AIM-Back study. Telehealth providers had flexibility in scheduling sessions and determining call durations which helped establish rapport and allowed Veterans to share their pain-related narratives without time constraints. This arrangement facilitated a more comprehensive and nuanced understanding of the Veterans’ experiences, fostering a stronger patient-PT working alliance. AIM-Back telehealth providers were also trained to use MI in their interactions with Veterans. Through the use of MI techniques emphasizing open-ended questions, affirmations, reflections, and summarizations, Veterans were guided in defining and evaluating personalized, values-based goals over the course of care. This approach was used to foster Veterans' intrinsic motivation to actively engage with home-based exercise and pain coping programs, potentially enhancing adherence.

### Culturally-competent care

Culture, which includes shared beliefs and behaviors, communication styles, views of roles and relationships, values, and traditions (72); impacts a person's pain experience (73). Culturally competent care effectively provides services that meet the social, cultural, and linguistic needs of patients (74), and is essential for reducing health disparities (75). Cultural competence applies to racial/ethnic minoritized communities and other communities that experience disparities including persons from low income, rural and Veteran communities (75). Culturally competent care is not achieved by merely having providers with similar backgrounds as their patients. Providers need to be aware of their own cultural perspective because ineffective communication, stereotyping or biased care can arise when cultural differences between patient and provider are unrecognized (76).

With respect to cLBP, cultural factors can impact when and how an individual chooses to seek care, their beliefs about the likely cause of pain, locus of control, self-efficacy, preferred coping strategies and receptivity to particular interventions (77, 78). For example, a core component of evidence-based PT for cLBP is helping patients adopt active coping for self-management, but some cultural perspectives may favor more passive strategies (e.g., rest, prayer, etc.) (79). Cultural considerations can also impact patients' trust in healthcare, their willingness to participate in PT, or enroll in clinical research studies (80). Helping potential participants understand clinical research and develop strong interpersonal connections with a PT may be especially important to build trust with persons in underserved communities (81). In the BeatPain study, participants considering enrollment have the opportunity to consult the study's web page or connect with a PT who can answer additional questions about participation.

Many BeatPain participants are persons of Hispanic/Latino ethnicity. Persons of Hispanic ethnicity living in the U.S represent an array of cultural sub-groups based on region of origin, degree of acculturation, socioeconomic status and other factors (82). Some similarities among persons of Hispanic ethnicity have been identified (83–85), and tend to be reflected in patients participating in the BeatPain study. These include approaching chronic pain with greater stoicism and a focus on continuing in social roles, particularly familial roles. BeatPain also serves people in rural communities who are more likely to use passive pain coping strategies such as heat/cold application, medication, etc. (12). Integrating MAPS and MI into BeatPain protocols has facilitated the delivery of culturally competent PT care. This may reflect MI's emphasis on open questions and reflective summaries that communicate respect for the patient's perspective on the causes and consequences of pain. The non-judgmental, patient-centered MI approach may reduce risks of misconceptions or implicit bias on the part of PTs (86). The goal-setting and problem-solving aspect of MAPS helps PTs center on activities of importance to the patient such as familial responsibilities. BeatPain PTs receive additional training in culturally competent care to help them examine their own cultural background and understand how it may interact with their patient's background to impact care (87), and to help PTs consider major cultural issues they may encounter (88). Initial training includes about 2 h for cultural competence and 10 h for MAPS. Ongoing training involves weekly meetings to discuss cases and quarterly peer-practice sessions.

The AIM-Back study serves Veterans, a community with unique experiences and a distinct culture that encompasses persons whose cultural background is further informed by gender, race, ethnicity etc. (24). During the design phase of AIM-Back, a partner engagement process with the Veteran community was incorporated to provide opportunities for patients, caregivers, clinicians, and administrative leaders to voice their views on the planning, implementation, and evaluation of the proposed care pathways. Attention was paid to gaining diverse input based on Veterans' race/ethnicity, gender and deployment experiences (89).

## Discussion

Although cLBP disproportionately impacts persons in minoritized, low income, Veteran and rural communities, clinical trials historically focus recruitment on urban, academic medical centers serving persons with greater economic resources and less diversity (90). Inclusion of patients from underserved communities is critical for all research, particularly for PCTs that recruit participants as part of routine care. Thus, PCTs risk reproducing and reinforcing disparities if they focus on urban, academic healthcare systems and fail to make intentional efforts to include persons from communities that experience disparities (91, 92).

Access to evidence-based, nonpharmacologic care is a likely contributor to pain prevalence and outcome disparities. Telehealth is an attractive strategy to mitigate access barriers in communities where nonpharmacologic providers are often unavailable. Data emerging from the COVID pandemic, however, raise concerns that the communities with the greatest need for improved access may not realize the benefits of telehealth without intentional efforts. Several studies suggest that persons from racial/ethnic minorized and/or rural communities, and those with lower income may be less likely to use telehealth (93–96).

Communities that experience disparities have been referred to as “evidence vacuums” because they are generally omitted from clinical research (92). Interventions found effective in urban, academic settings cannot be presumed effective for persons in underserved communities. Although clinical trials support equivalence between telehealth and in-person PT (97), these trials have not focused on patients in underserved communities (92). Emphasis on telehealth as a strategy to overcome disparities could paradoxically have the opposite effect if the challenges for providing care to persons in underserved communities are not reflected in research (98). This concern motivated the BeatPain and AIM-Back studies to partner with CHCs and mostly non-academic VA facilities respectively. This paper describes the important step of tailoring the PT interventions to meet the unique needs of patients served in these settings. While these PCTs are ongoing, we believe our experiences can inform future efforts to use telehealth to make effective nonpharmacologic pain care accessible for persons with cLBP in underserved communities.

## Conclusion

In order to address disparities in health and health care, pragmatic clinical trials should include patients in communities that are historically under-represented in research. Inclusion of settings and participants who are familiar with pragmatic research requires careful attention to unique challenges when developing interventions and implementation strategies.

## Data Availability

The original contributions presented in the study are included in the article/supplementary Materials, further inquiries can be directed to the corresponding author.
